# Comparative analysis and gut bacterial community assemblages of grass carp and crucian carp in new lineages from the Dongting Lake area

**DOI:** 10.1002/mbo3.996

**Published:** 2020-03-16

**Authors:** Sheng Zou, Liang Gong, Tahir Ali Khan, Lifei Pan, Liang Yan, Dongjie Li, Lina Cao, Yanping Li, Xuezhi Ding, Ganfeng Yi, Yunjun Sun, Shengbiao Hu, Liqiu Xia

**Affiliations:** ^1^ State Key Laboratory of Developmental Biology of Freshwater Fish, Hunan Provincial Key Laboratory of Microbial Molecular Biology College of Life Science Hunan Normal University Changsha China

**Keywords:** connectivity of bacteria, crucian carp, Dongting Lake area, grass carp, gut microbiota, high‐throughput sequencing

## Abstract

Gut microbiota are known to play an important role in health and nutrition of the host and have been attracting an increasing attention. Farming of new lineages of grass carp and crucian carp has been developed rapidly as these species were found to outperform indigenous ones in terms of growth rate and susceptibility to diseases. Despite this rapid development, no studies have addressed the characteristics of their gut microbiota as a potential factor responsible for the improved characteristics. To reveal whether microbiomes of the new lineages are different from indigenous ones, and therefore could be responsible for improved growth features, intestinal microbiota from the new lineages were subjected to high‐throughput sequencing. While the phyla Firmicutes, Fusobacteria and Proteobacteria were representing the core bacterial communities that comprised more than 75% in all fish intestinal samples, significant differences were found in the microbial community composition of the new linages versus indigenous fish populations, suggesting the possibility that results in the advantages of enhanced disease resistance and rapid growth for the new fish lineages. Bacterial composition was similar between herbivorous and omnivorous fish. The relative abundance of Bacteroidetes and Actinobacteria was significantly higher in omnivores compared to that of herbivores, whereas Cetobacterium_sp. was abundant in herbivores. We also found that the gut microbiota of freshwater fish in the Dongting lake area was distinct from those of other areas. Network graphs showed the reduced overall connectivity of gut bacteria in indigenous fish, whereas the bacteria of the new fish lineage groups showed hubs with more node degree. A phylogenetic investigation of communities by reconstruction of unobserved states inferred function profile showed several metabolic processes were more active in the new lineages compared to indigenous fish. Our findings suggest that differences in gut bacterial community composition may be an important factor contributing to the rapid growth and high disease resistance of the new fish lineages.

## Graphical abstract

Our results are the first to reveal the gut microbial composition in the new lineages of grass carp and crucian carp from the Dongting lake area and differences of gut microbiota between new lineages and the indigenous fish populations. These findings may offer an understanding why the new lineages could grow faster and possess higher disease resistance. We also concluded that the gut microbiota of freshwater fish in the Dongting lake area was distinct from those of other areas.

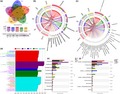

## 1 INTRODUCTION

Intestinal microbiotas, which provide several biological functions in the host, including health maintenance, immune regulation, and nutrition utilization, comprise all the microorganisms living in the intestine of animals and their genetic material (Grond, Lanctot, Jumpponen, & Sandercock, 2017; Liu et al., 2016; Ray, Ghosh, & Ringo, 2012; Yang et al., 2017). Various culture‐independent techniques (e.g., temporal temperature gradient electrophoresis [TTGE], denaturing gradient gel electrophoresis [DGGE], fluorescent in situ hybridization [FISH], and OMIC approaches) are recently available to analyze microbiota, determine the microbial assembly abundance of a particular taxa, and assess bacterial–host interactions (Dueñas et al., 2015; Egerton, Culloty, Whooley, Stanton, & Ross, 2018). Next‐generation high‐throughput sequencing is the latest method for analyzing microbiota, improving our understanding of diverse ecosystem of host‐associated microbes (Li, Liu, et al., 2018).

Fish originated over 600 million years ago (Egerton et al., 2018), and over three billion people worldwide depend on fish for at least 20% of their protein intake and approximately 20 kg of fish is consumed per capita per annum (Food & Agriculture Organisation of the United Nations [FAO], 2016). Even though research on the fish gut microbiota started in the early half of the 20th century, previous studies have not documented the characteristics of the gut microbiota of new lineages, such as Pure‐line grass carp (*Ctenopharynodon idellus*), Hefang crucian carp (*Carassius auratus*), and Xiangyun crucian carp (*C. auratus*) from the Dongting lake area. The research work related to the gut microbiota of fish is dwarfed by that on humans and mammals. The colonization of fish intestinal microorganisms is an extremely complex process because of the influence of fish species, water environments, seasonal changes, and other factors (Ni, Yu, Zhang, & Gao, 2012; Qin et al., 2016; Ye, Amberg, Chapman, Gaikowski, & Liu, 2014). Previous studies focused mainly on pattern of bacterial composition of wild‐type fish species, for example, zebrafish (Liu et al., 2016; Siriyappagouder et al., 2018), common carp (Chang et al., 2019), bighead carp (Li, Zhu, et al., 2018), Atlantic salmon (Klemetsen, Willassen, & Karlsen, 2019), Atlantic cod (Walter, Bagi, & Pampanin, 2019), rainbow trout (Etyemez & Balcázar, 2015), blunt snout bream, and topmouth culter (Li, Liu, et al., 2018).

Farming of grass carp and crucian carp is an important economic pillar in the Dongting Lake area of Hunan Province (China). The grass carp, an herbivorous freshwater fish, made it be the largest freshwater aquaculture product in China, which the production reached 5.7 million tons in 2015 (Lin et al., 2018). The Pure‐line grass carp is a new grass carp lineage that has the advantages of strong disease resistance and rapid growth, which was developed through gynogenesis using ultraviolet‐treated heterologous sperm to activate the eggs of grass carp and subsequently cold shock or heat shock treatment on eggs to double the chromosomes (Mao et al., 2019; Zhang et al., 2011). The crucian carp as an omnivorous freshwater fish is one of the most important and popular freshwater aquaculture species in China, in which its total yield is over 3 million tons per year (Wu et al., 2019). Crucian carp is rich in nutrients such as the vitamin, protein, unsaturated fatty acids, and inorganic components (Liu, Sha, Wang, Li, & Bureau, 2018). New species of crucian carps, such as Hefang crucian carp and Xiangyun crucian carp, are hybrids of other species of fish. Hefang crucian carp derived from Japanese white crucian carp (♀) × red crucian carp (♂), which exists the advantages of a high survival rate, strong resistance, and a fast growth rate. Xiangyun crucian carp that is a kind of allotriploid crucian carp derived from diploid Japanese white crucian carp (♀) × allotetraploid hybrids (♂) (Wang et al., 2019). Previous reports validate sterile hybrids, involved in Pure‐line grass carp and Xiangyun crucian carp, and have some advantages such as faster growth (~1.43 times) and higher anti‐disease ability (~1.5 times) (Chen et al., 2009; Liu et al., 2001; Xiao et al., 2019). Japanese white crucian carp (*Carassius auratus curvier*) are characterized by strong reproductive ability and rapid growth rates (Liu et al., 2019). Both Hefang crucian carp and Xiangyun crucian carp inherit superiority of white crucian carp. All of these freshwater fish are economically very important because of their high nutritional value and are widely distributed in the lakes and reservoirs of China. The present study investigated the intestinal microbial structures of the five groups of fish from Dongting lake area to assess the relationship among genetic differentiation, feeding habits differences, and gut microbiota structure.

## 2 EXPERIMENTAL PROCEDURES

### 2.1 Fish and sample collection

**Figure A1:**
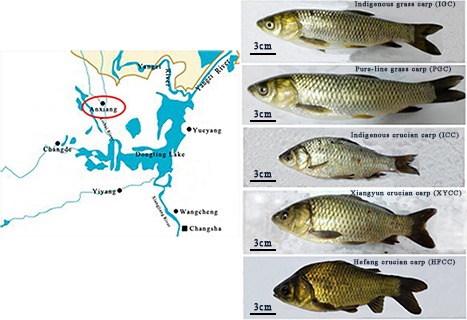
Areas (red ellipse) from which fish gut samples were collected: Hunan province, China (Left), and the new fish lineages: grass carp and crucian carp, with  their indigenous species

Five species of fish (five individuals each), nearly half year old and no visible disease symptoms, were obtained from intensive cultivation fish ponds of Anxiang county (N29°19′12″, E111°25′12″) in Dongting Lake area (N27°39′–29°51′, E111°19′12″–113°34′12″) (Figure A1), which include Pure‐line grass carp (PGC), Indigenous grass carp (IGC), Xiangyun crucian carp (XYCC), Hefang crucian carp (HFCC), and Indigenous crucian carp (ICC) (Table 1). Experimental fish were euthanized by overdose of tricaine methane sulfonate (MS222; 50 mg/L) and were aseptically dissected to obtain midgut samples and their contents (Cordova & Braun, 2007; Li, Liu, et al., 2018; Song et al., 2016). Samples were stored at −80°C until further processing.

**Table 1:** Details of sample collection

Fish species	Number of samples	Collecting seasons	Length (cm)	Weight (g)
Pure‐line grass carp	DKC 1–5	Autumn	26.63 ± 5.33	333.01 ± 14.48
Indigenous grass carp	DCC 1–5	Autumn	24.98 ± 4.19	305.90 ± 22.53
Xiangyun crucian carp	DXJ 1–5	Autumn	16.05 ± 8.25	433.02 ± 37.91
Hefang crucian carp	DHF 1–5	Autumn	15.61 ± 4.73	423.31 ± 40.06
Indigenous crucian carp	DJY 1–5	Autumn	13.86 ± 6.72	372.45 ± 35.19

### 2.2 DNA extraction and high‐throughput 16S rRNA amplicon sequencing

Microbial DNA was isolated from the fish gut contents samples using PowerFecal™ DNA Isolation Kit (Mobio), following the manufacturer's instructions. The V4 hypervariable region of the 16S rRNA gene was amplified, sequenced, and analyzed to define composition of the bacterial community. Amplification primers were generated with the following PCR primers of 515F (5′‐GTGCCAGCMGCCGCGGTAA‐3′) and 806R (5′‐GGACTACHVGGGTWTCTAAT‐3′) (Caporaso et al., 2011), Polymerase chain reaction (PCR) experiments (30 μl) of each sample in triplicate were also prepared, each containing approximately 15 μl Phusion Master Mix (2×), 3 μl Primer (2 μM), 10 μl DNA (1 ng/μl), and 2 μl H_2_O (Gao et al., 2019). PCR amplification was conducted using the following thermocycles: initial denaturation at 98°C for 1 min; 30 cycles at 98°C for 10 s, 50°C for 30 s, and 72°C for 30 s; and a final extension at 72°C for 5 min. Equal amounts of each sample were combined and gel‐purified using a QIAquick Gel Extraction Kit (QIAGEN) before being quantified using PicoGreen (Mardis & McCombie, 2017). All PCR products were then sequenced using on an Illumina HiSeq 2500 platform, and 250 bp paired‐end reads were generated (Caporaso et al., 2012) at Novogene Bio‐informatic Biotechnology Co., Ltd.

### 2.3 Quality control and processing

The sequence length of the V4 hypervariable region of 16S rRNA gene was approximately 250 base pairs for most microbial species. All paired‐end reads from the original DNA fragment were assigned to each sample according to the unique barcodes and then merged using FLASH (V1.2.7, http://ccb.jhu.edu/software/FLASH/) to obtain raw sequences (Magoc & Salzberg, 2011). Prior to getting the quality‐filtered reads, removed chimeric sequences in clean sequences using UCHIME algorithm software (http://www.drive5.com/usearch/manual/uchime_algo.html) (Edgar, Haas, Clemente, Quince, & Knight, 2011) and Gold database (http://drive5.com/uchime/uchime_download.html), low‐quality raw sequences were strictly filtered to generate clean sequences by QIIME software (Quantitative Insights into Microbial Ecology, version 1.7.0, http://qiime.org/scripts/split_libraries_fastq.html) (Bokulich et al., 2013). Sequence data were deposited in the NCBI Sequence Read Archive (SRA) (Accession number: SRR9645031, SRR9645032, SRR9645033, SRR9645034, SRR9645035). The data were analyzed on the free online platform of Majorbio I‐Sanger Cloud Platform (http://www.i-sanger.com).

### 2.4 Sequencing data analysis

The effective sequences were clustered to operational taxonomic units (OTUs) at 97% sequence similarity by Usearch (version 7.0, http://drive5.com/uparse/) (Edgar, 2010), after which taxonomic annotation was conducted using the RDP classifier (Ribosomal Database Project, version 2.2, http://sourceforge.net/projects/rdp-classifier/) at a 70% confidence level. Alpha‐diversity indices (bacterial community richness: Chao; bacterial community diversity: Shannon) based on the OTU level abundance profile were determined using the Mothur software (version 1.30.1, http://www.mothur.org/wiki/Schloss_SOP#Alpha_diversity) at 97% sequence similarity (Quast et al., 2013; Wang, Garrity, Tiedje, & Cole, 2007). As beta‐diversity analysis, principal coordinates analysis (PCoA) based on unweighted‐uniFrac distances was performed using the Vegan package in R software (Chi et al., 2019). Linear discriminant analysis effect size (LEfSe) was used to examine the differentially abundant bacterial communities of the groups by LefSe software (http://huttenhower.sph.harvard.edu/galaxy/root?tool_xml:id=lefse_upload). Analysis of similarity statistics (ANOSIM) was estimated using the Bray–Curtis distance matrix to test the significance of differences between the five groups of fish (Douterelo, Sharpe, & Boxall, 2013). One‐way ANOVA and a two‐tailed Student's *t* test were applied to determine differences in intestinal bacterial communities between the five fish groups (Li, Liu, et al., 2018); *p*‐value <.05 and *p*‐value <.01 were considered statistically significant and highly significant, respectively.

### 2.5 Microbial network construction and prediction for the potential function of the OUTs sequences

A network consisting of non‐random co‐occurrences was constructed according to a Spearman correlation coefficient >0.5 in the R platform without considering the compositional variation (Chen et al., 2018). Phylogenetic investigation of communities by reconstruction of unobserved states (PICRUSt, version 1.0) was used to predict the functional content based on the 16S rRNA sequencing data (Wong et al., 2016).

## 3 RESULTS

### 3.1 Sequencing quality

**Figure A2:**
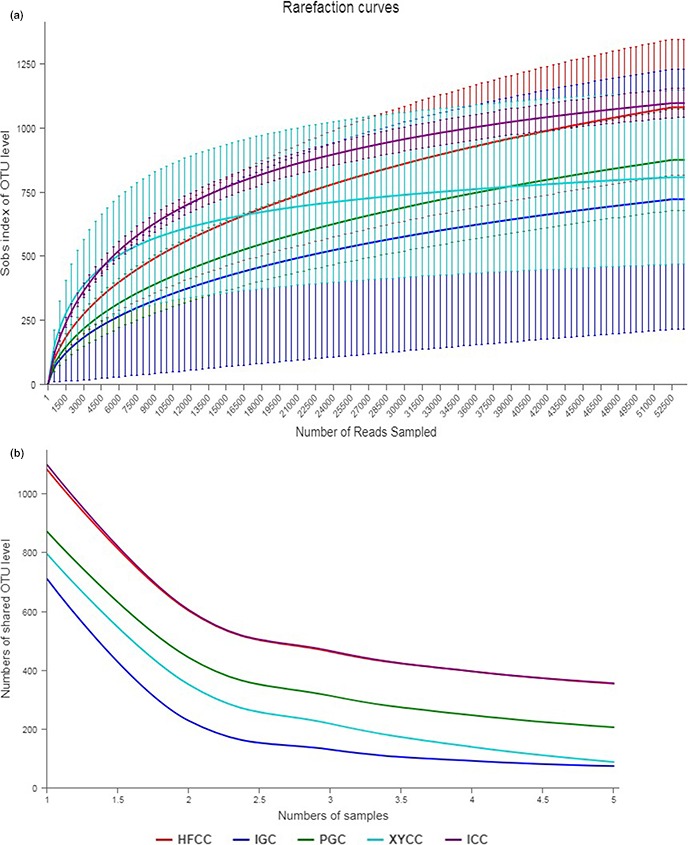
Assessment of data quality. (a) Rarefaction curves. (b) Core curves based on the 16S rRNA gene reads OTUs

We obtained the 16S rRNA V4 amplicon sequences of the bacterial communities of 25 intestine samples from five fish species. After the adapter and quality trimming of 1,915,354 raw sequences, a total of 1,789,385 effective sequences were clustered into 4,896 OTUs (Table S1 at https://doi.org/10.6084/m9.figshare.11389638.v2). These OTUs were assigned to 48 phyla, 122 classes, 255 orders, 480 families, 1,123 genera, and 2,130 species (Table S2 at https://doi.org/10.6084/m9.figshare.11389638.v2). The rarefaction curves based on Sobs index at 97% identity threshold reached a saturation phase at approximately 750 OUTs, indicating that the sequencing data were reasonable and could reliably describe the full microbial diversity (Figure A2a). The core species curves were horizontal when the number of samples was greater or equal to 4, thereby allowing us to capture most of the bacterial communities (Figure A2b). Hence, all sequences may contribute to a complete and even representation of commensal microbiome and are considered to be appropriate for further analysis.

### 3.2 Alpha‐ and beta‐diversity analysis

**Figure 1:**
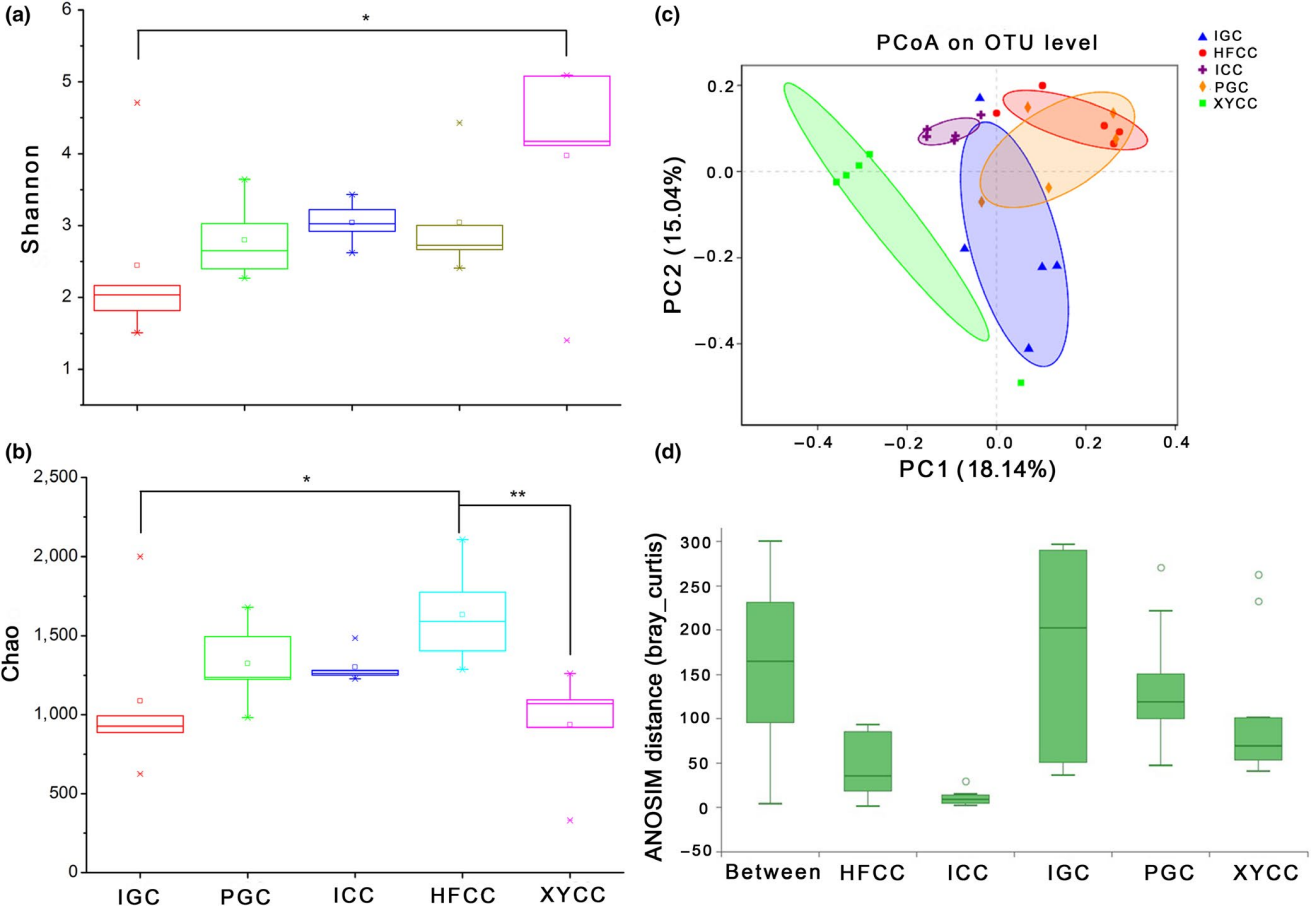
Diversity analysis of gut microbiota in the five fish groups. (a) Microbial diversity (alpha diversity) was estimated according to Shannon index. (b) Bacterial community richness (alpha diversity) was assessed by Shannon index. (c) PCoA (beta diversity) showed the separated distribution of bacterial community composition in all individuals. (d) ANOSIM (beta diversity) estimated at the OTUs level in the five groups of fish samples. *p < .05, **p < .01

**Figure A3:**
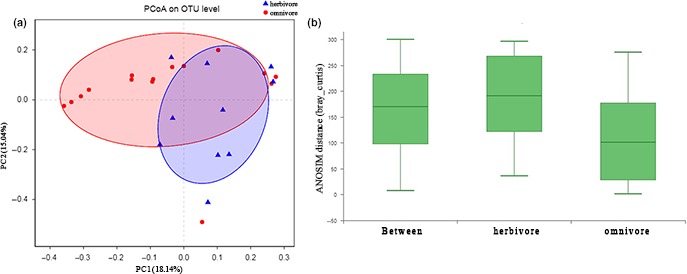
Analysis of bacterial community composition between herbivore and omnivore. (a) PcoA and (b) ANOSIM showed the differences in the bacterial community composition in all individuals

The alpha diversity of the microbial community of the five fish groups was expressed using the Shannon and Chao indexes (Figure 1a,b). The microbial diversity of XYCC group was highest according to Shannon index and differed significantly between XYCC and IGC groups (*p* < .05). Bacterial community richness of HFCC group was highest according to Chao index, in which it was significantly higher than that of the IGC and XYCC groups (*p* < .05). Additionally, microbial communities from IGC group displayed lower diversity and richness compared with the other four groups, namely PGC, ICC, HFCC, and XYCC. PCoA based on Bray–Curtis dissimilarity, which revealed a clear clustering pattern, was used to examine the relationship between the microbial communities across different fish species (Figure 1c). The resulting bacterial compositions of each fish lineages were clearly separated by PC1 and PC2, such as no cross‐link among three crucian carp (HFCC, XYCC, and ICC) as well among two grass carp (PGC and IGC), respectively. ANOSIM showed significant differences in microbial community structure among the five fish species (OTU level: *R* = .44 and *p* < .001) (Figure 1d), in which significant differences between new lineages and indigenous fish (IGC vs. PGC—*p* < .05, ICC vs. HFCC—*p* < .01, ICC vs. XYCC—*p* < .01) were revealed during pairwise comparisons. The bacterial community composition between herbivorous grass carp and omnivorous crucian carp was also evidently distinct (OTU level: R = 0.22 and *p* < .05) (Figure A3).

### 3.3 Microbiota composition analysis

**Figure 2:**
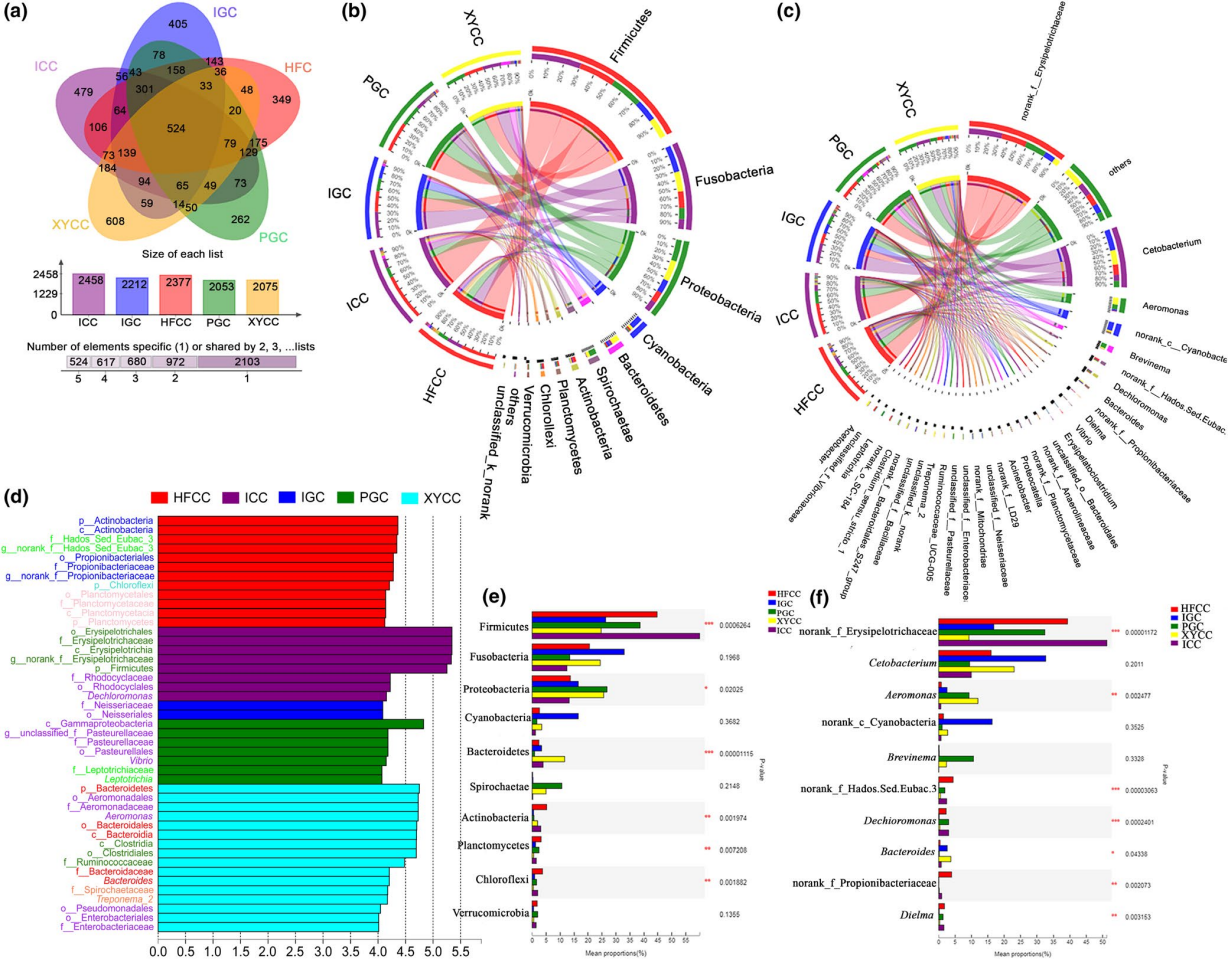
Taxonomic composition of the gut microbiota in the five fish groups. (a) Venn diagram showed the shared and unique OTUs in all fish samples. (b) Distribution of microbial community was visualized by Circos for each fish sample at phylum level. (c) Distribution of microbial community was visualized by Circos for each fish sample at genus level. (d) LEfSe was applied to finding the differential abundance using a cutoff of 4 and a significance threshold of *p* < .05. *Y*‐axis labels are color‐coded for different bacterial taxa: Firmicutes—dark green, Proteobacteria—purple, Bacteroidetes—red, Actinobacteria—blue, Fusobacteria—green, Planctomycetes—pink, Spirochaetes—coral, and Chloroflexi—cyan. (e) Statistical comparison of the relative abundance the five fish groups by one‐way ANOVA at phylum level. (f) Statistical comparison of the relative abundance the five fish groups by one‐way ANOVA at genus level. *p < .05, **p < .01, ***p < .001

**Figure A4:**
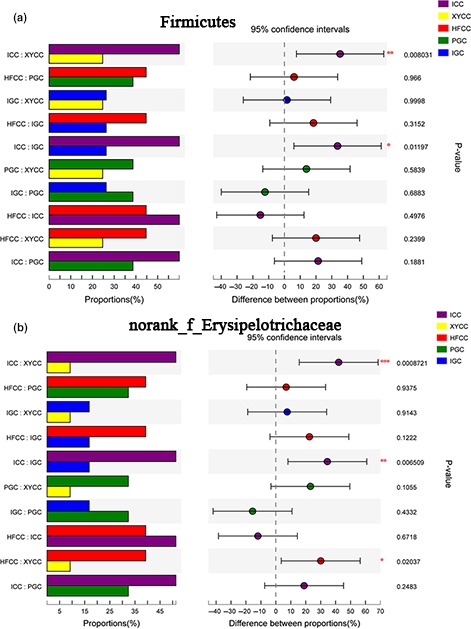
One‐way ANOVA showed relative abundance differences of (a) Firmicutes and (b) *Erysipelotrichaceae* in any two groups of fish. *p < .05, **p < .01, ***p < .001

Given that the microbiota community structure of the five fish groups exhibited significant differences, we further explored the bacterial community composition of each fish group. The results of Venn diagram showed that the unique OTUs of each fish group were within the range from 262 to 608 and represented the differentiations in the intestinal microbial composition. The Venn diagram identified 524 OTUs shared among all five groups and represented the core microbiota of grass carp and crucian carp (Figure 2a). The predominant bacterial components from all of the gut samples were similar at the phylum level, but their differences were due to the different proportions of these core bacteria (Figure 2b). Firmicutes, Fusobacteria, Proteobacteria, Cyanobacteria, Bacteroidetes, Spirochaetae, Actinobacteria, Planctomycetes, Chloroflexi, and Verrucomicrobia were the top‐10 dominant phyla at the >1% abundance and occurred in all the fish groups. Notable differences were found in these fish groups' microbial communities, including Firmicutes, Proteobacteria, Bacteroidetes, Actinobacteria, Planctomycetes, and Chloroflexi (*p* < .05, one‐way ANOVA) (Figure 2e). Among these communities, the relative abundance of Firmicutes was higher in the ICC group than in rest groups (ICC_59.97% vs. HFCC_44.71%, IGC_26.31%, PGC_38.63%, XYCC_24.70%), and the ratios of these bacterial taxa in ICC (59.97%) differed significantly from those in XYCC (24.70%, *p* < .01, one‐way ANOVA) and IGC (26.31%) (*p* < .05, one‐way ANOVA) (Figure A4a). At the genus level, norank_f_Erysipelotrichaceae was the most abundant in all fish groups, followed by *Cetobacterium*, *Aeromonas*, *Brevinema*, *Dechioromonas*, *Bacteroides*, *Dielma*, and the unidentified genera of the family Hados.Sed.Eubac.3 and Propionibacteriaceae (Figure 2c). In total, the differences of relative abundances were more evident for these genera of norank_f_Erysipelotrichaceae, *Aeromonas*, norank_f_Hados.Sed.Eubac.3, *Dechioromonas*, *Bacteroides*, and *Dielma* at different fish groups (*p* < .05, one‐way ANOVA) (Figure 2f). Interestingly, norank_f_Erysipelotrichaceae genus, classified into Firmicutes, was significantly higher in ICC group (51.30%) as compared to the XYCC group (9.23%, *p* < .001, one‐way ANOVA) and IGC group (16.82%, *p* < .01, one‐way ANOVA), which indicated that norank_f_Erysipelotrichaceae genus played a critical role in Firmicutes due to the higher relative abundance of Firmicutes in the ICC group than that in XYCC and IGC (Figure A4b). To explore the variation of the microbial community composition in different fish groups, we performed LEfSe analysis to detect differences in the relative abundance of bacterial taxa (Figure 2d). It was noteworthy that the number of taxa identified in XYCC group (16 taxa) was higher than that in other four groups (12, 8, 2, and 7 taxa in HFCC, ICC, IGC, and PGC, respectively), and Firmicutes, Proteobacteria (mainly class Gammaproteobacteria), Bacteroidetes, and Spirochaetae were the most abundant taxa in XYCC group. In addition, some taxa belonging to Actinobacteria and Planctomycetes were the overrepresented taxon in HFCC group, whereas *Erysipelotrichaceae* (Firmicutes) and *Rhodocycclaceae* (Proteobacteria, Betaproteobacteria) were observed as the significantly abundant type in the ICC group. Although bacteria belonging to the phylum Proteobacteria in grass carp were dominant taxa, the class Betaproteobacteria (with Neisseriaceae as the representative) was largely abundant in the IGC group. By contrast, the predominant taxon was Gammaproteobacteria in the PGC group.

To further validate the relationship between genetic differentiation and gut microbiota structure, the data analysis revealed the gut microbiota of fish had significant difference after genetic differentiation, albeit their kinship. The bacterial composition between PGC and IGC exhibited significant difference. The class Betaproteobacteria (with Neisseriaceae as the representative) was largely abundant in the IGC group, but the predominant taxon was Gammaproteobacteria in the PGC group. Additionally, both Hefang crucian carp and Xiangyun crucian carp have a common parent of white crucian carp, and the bacterial composition between HFCC and XYCC has notable difference. Bacterial community richness of HFCC group was significantly higher than that of the XYCC groups. The phylum Actinobacteria in the HFCC group was predominant taxon, whereas the phylum Bacteroidetes in the XYCC group was superior taxon.

**Figure 3:**
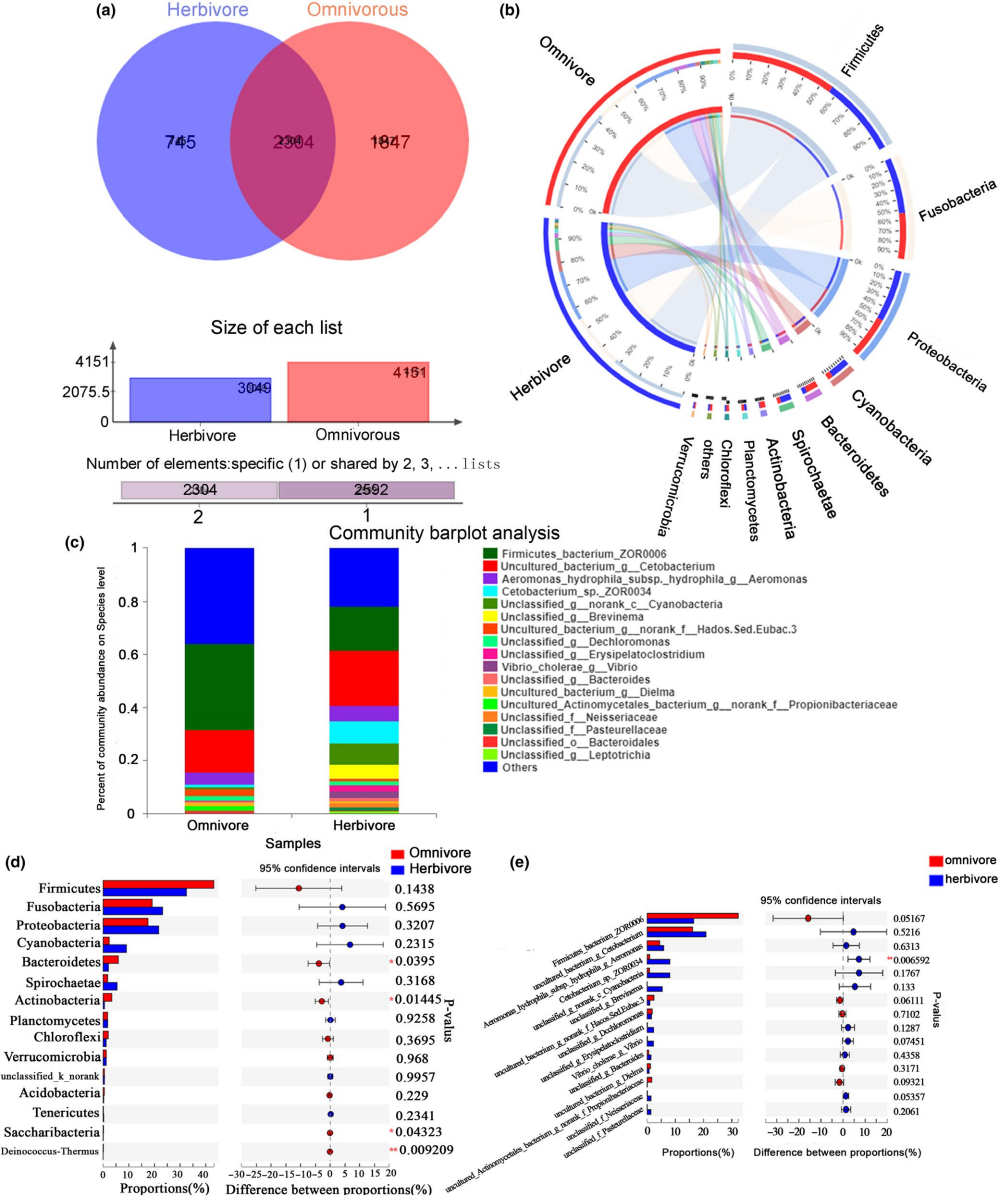
Comparative analysis of gut microbiota between herbivore (grass carp) and omnivore (crucian carp). (a) Venn diagram showed shared and unique OTUs between herbivore and omnivore. (b) Distribution of microbial community was visualized by Circos for herbivore and omnivore at phylum level. (c) Relative abundance of top‐15 gut microbial was visualized at species level. (d) Student's *t* test bar plot was employed to reveal statistical differences on phylum level of bacterial communities between herbivore and omnivore. (e) Student's *t* test bar plot was employed to reveal statistical differences on species level of bacterial communities between herbivore and omnivore. *p < .05, **p < .01

We further compared the characteristics of intestinal microbiota between herbivorous fish (IGC and PGC) and omnivorous fish (ICC, HFCC, and XYCC). The shared microbiome between the two feeding types comprised of 2,304 OTUs, while there were 745 and 1,847 unique OTUs in herbivore and omnivore, respectively (Figure 3a). Bacterial composition at phylum and species level in the two types of feeding habits of fish was similar (Figure 3b,c). However, the relative abundance of Bacteroidetes and Actinobacteria was significantly higher in omnivores compared with herbivores (*p* < .05, Student's *t* test) (Figure 3d). Furthermore, *Cetobacterium*_sp._ZOR0034 was more abundant in herbivores (*p* < .01, Student's *t* test) (Figure 3e).

### 3.4 Association network between bacterial community

**Figure 4:**
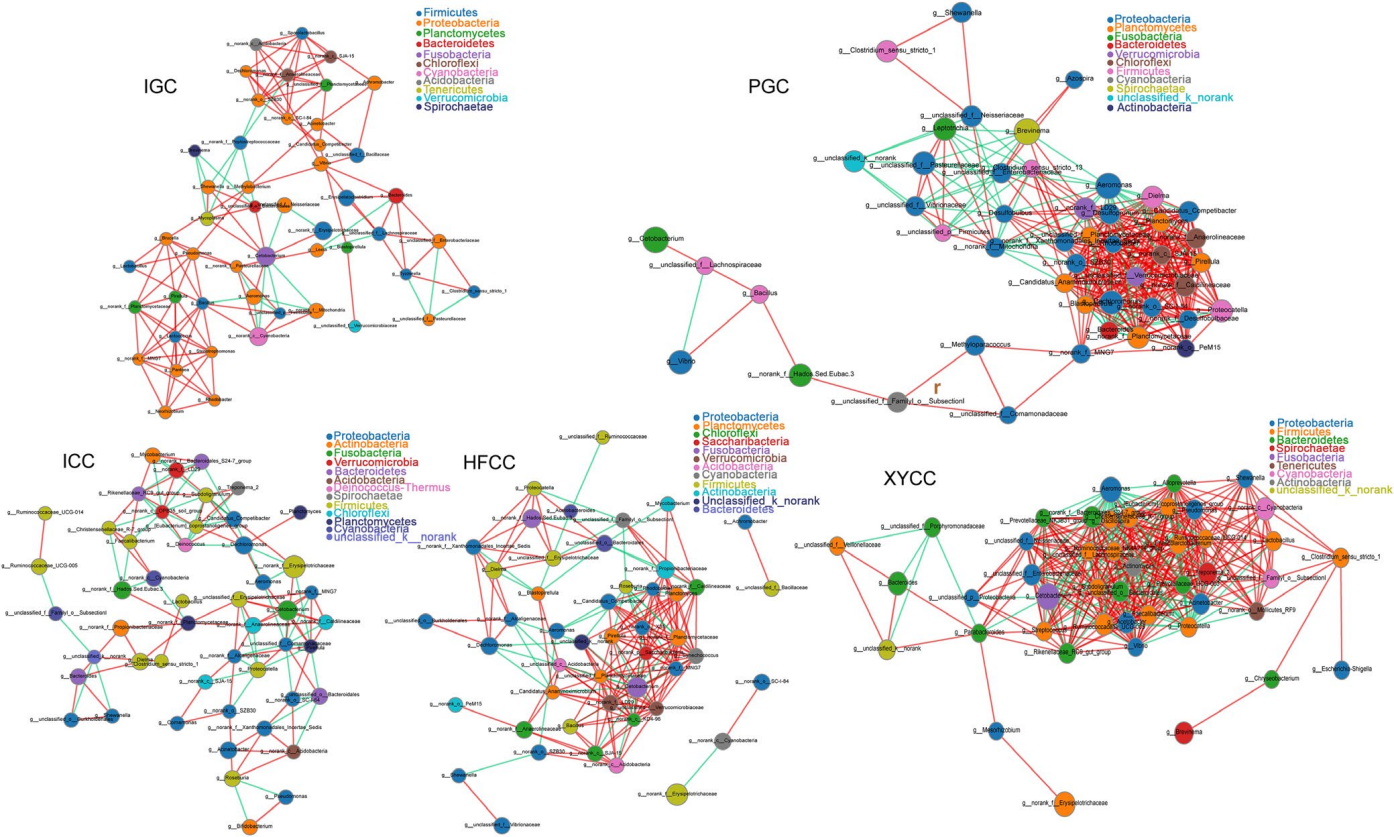
Network graphs of bacterial relationship in each fish group. Node color corresponds to phylum taxonomic classification. Edge color represents positive (red) and negative (green) correlations

**Figure 5:**
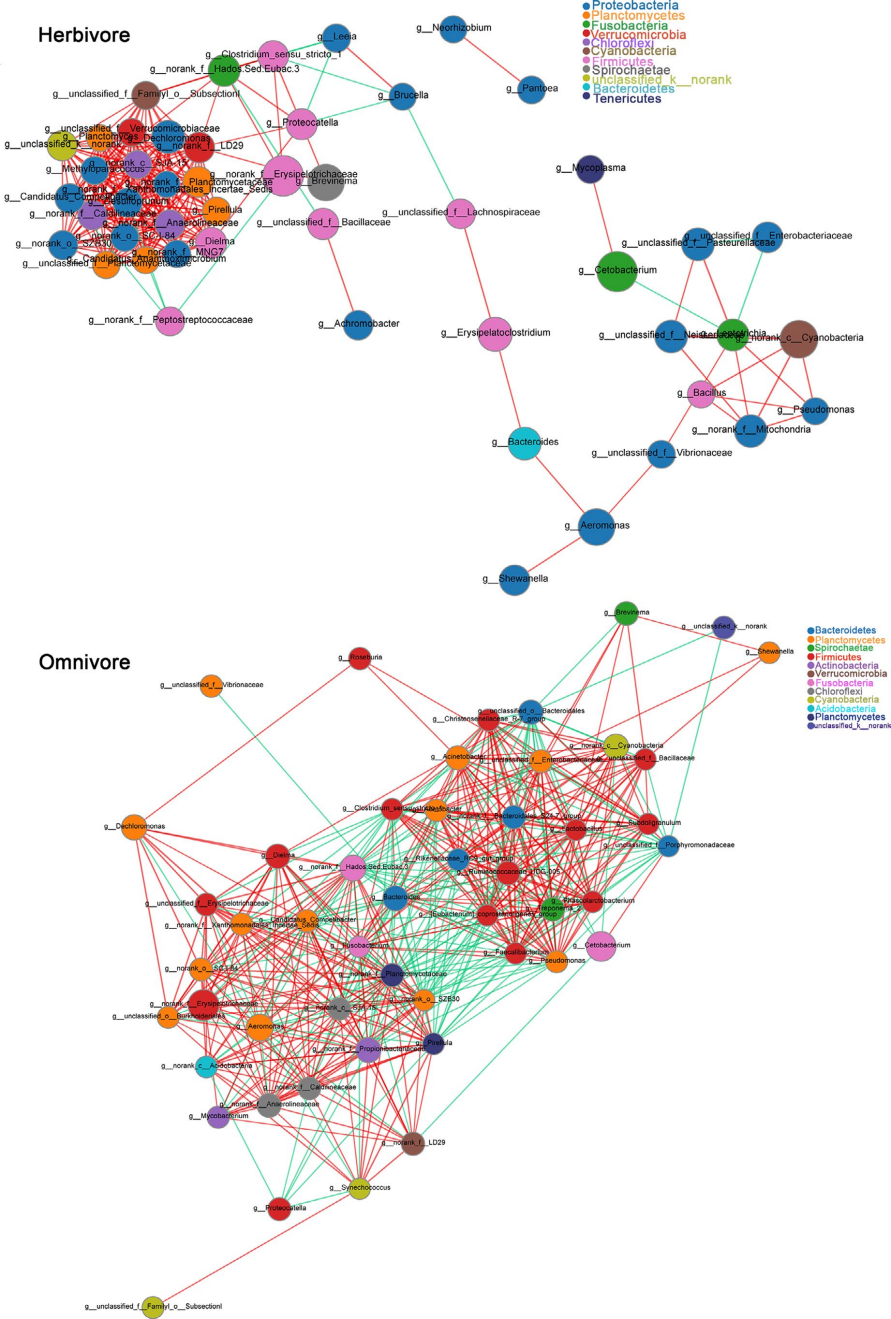
Network graphs of bacterial relationship in herbivore (grass carp) and omnivore (crucian carp), respectively. Node color corresponds to phylum taxonomic classification. Edge color represents positive (red) and negative (green) correlations

The co‐occurrence network analyses of top‐50 abundant intestinal bacteria in each fish group revealed that their interactive patterns exhibited remarkable distinctions, such as established positive and negative correlations based on the network plots were different (Figure 4). In addition, several nodes were negatively correlated with other nodes on the network, such as *Aeromonas*, *Cetobacterium*, norank_f_Erysipelotrichaceae, *Shewanella*, *Bacillus*, and *Bacteroides*, in which the patterns were caused by favorable genera for fish's survival outcompeting with less favorable ones. Similarly, the co‐occurrence network also showed that the bacterial interaction patters were different between herbivores and omnivores. The number of negatively correlated nodes were lower in herbivorous than in omnivorous fish (Figure 5). The majority of bacteria showed synergy to a large extent in herbivorous fish.

### 3.5 Functional prediction

**Figure 6:**
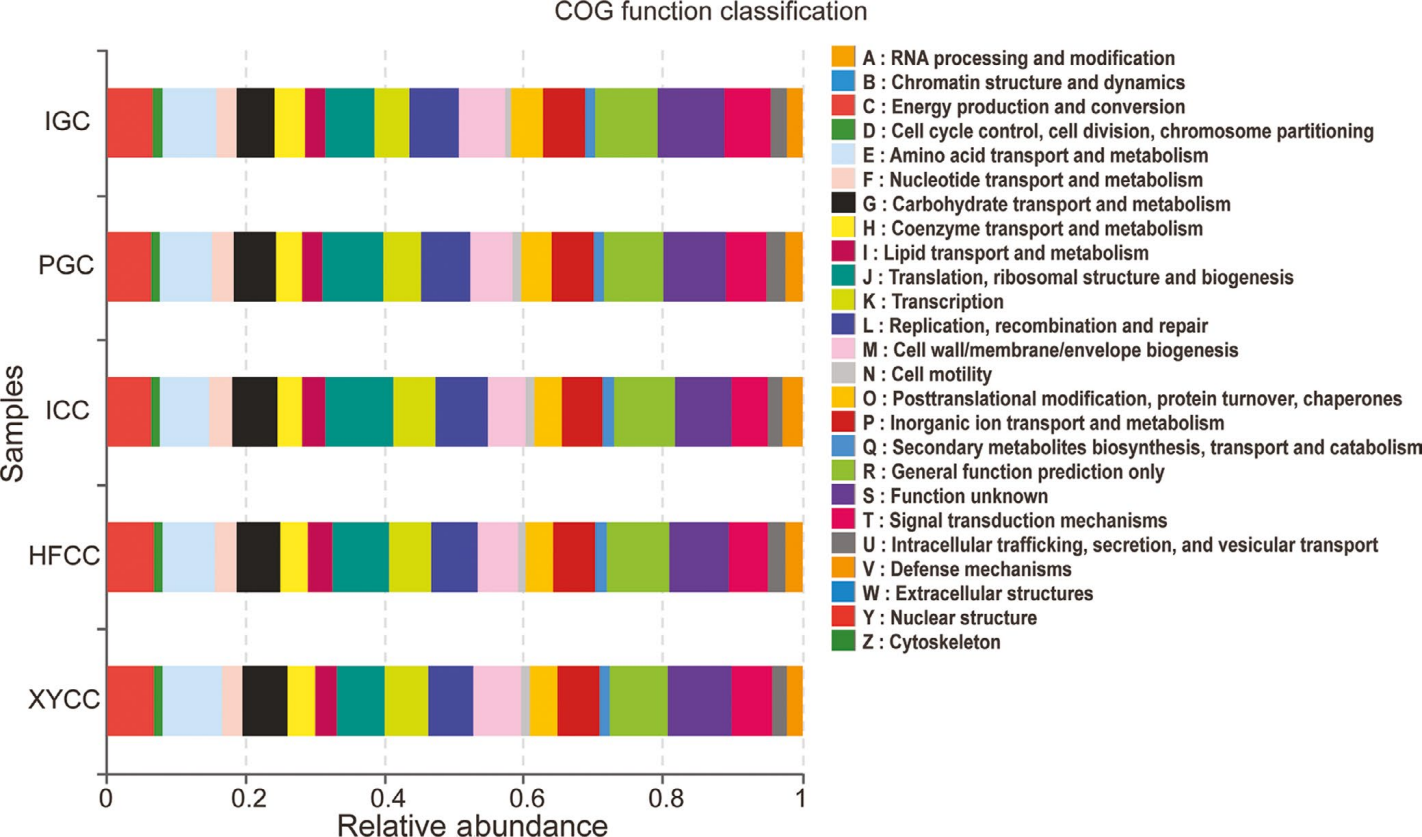
Clusters of Ortholog Groups (COG) function classification of gut microbiota in the five fish groups. The most clustered COG items were amino acid, carbohydrate, nucleotide, lipid, and coenzyme transport and metabolism

**Figure A5:**
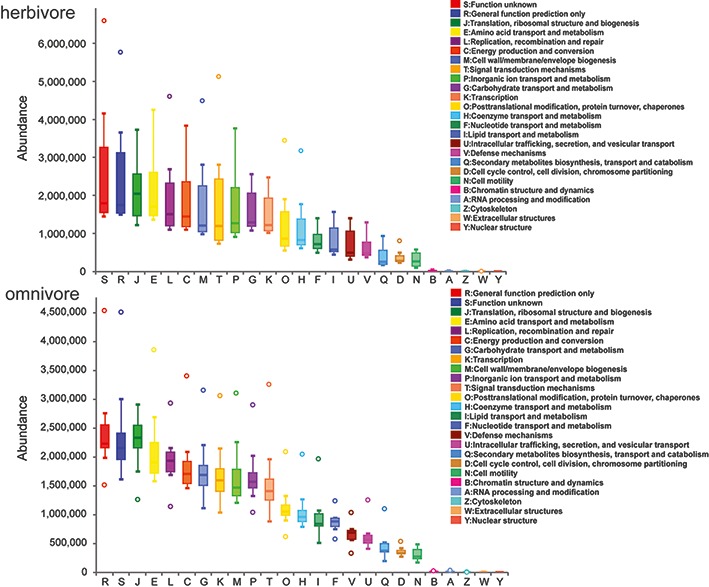
Potential function prediction of gut microbiota in both herbivore and omnivore fish

Phylogenetic investigation of communities by reconstruction of unobserved states‐predicted COG functional classification showed similar microbial functional features in every fish group, including amino acid transport and metabolism, energy production and conversion, carbohydrate transport and metabolism, nucleotide transport and metabolism, coenzyme transport and metabolism, lipid transport and metabolism, and signal transduction mechanisms (Figure 6). Interestingly, these bacterial functions in PGC (new lineage grass carp) were lower than that in IGC Indigenous grass carp). The aforementioned metabolic processes were more active in HFCC and XYCC (new lineage crucian carp) compared to ICC (indigenous crucian carp) (Table S3 at https://doi.org/10.6084/m9.figshare.11389638.v2). Predicted COG profiles also suggested great differences between the microbial processes potentially ongoing in herbivore and omnivore intestinal communities (Figure A5). The pathways involved in the transport and metabolism of carbohydrates, amino acids, nucleotides, lipids/ fatty acids, secondary metabolites, inorganic ion, and energy production and conversion were more prominent in the omnivorous fish gut microbiome compared with herbivorous fish. However, this microbial metabolism, such as coenzyme transport, cell wall/membrane/envelope biogenesis, and signal transduction mechanisms, tended to be more active in herbivorous fish. The results indicated gut microbial community not only played important roles in host metabolism, but also differentiated on the basis of diet and species.

## 4 DISCUSSION

Gut microbiota, which continues to be an intrinsic component of the host, can help host acquire the calories and nutrients present in various complex dietary and has attracted great attention from the scientific community in recent year (Costello, Gordon, Secor, & Knight, 2010; Li, Liu, et al., 2018). The establishment of the gut microbiota in fish is a complex process and a specific reflection of microorganisms in rearing water, diet, and the environment. The sort and number of microorganisms presented in fish are lower than that in warm‐blooded animals and fluctuate greatly with host age, nutrition, and environment (Riiser et al., 2019). In this study, the gut microbiota profiles in the two types of grass carps and three types of crucian carps were characterized and compared, subsequently describing the relationship among genetic differentiation, feeding habits differences, and gut microbiota structure.

According to previous reports, Firmicutes and Proteobacteria are dominant phyla in most of fish at the phylum level (Gong et al., 2019; Jiang et al., 2019; Li et al., 2013). Similar results were presented in our study, where Firmicutes, Fusobacteria and Proteobacteria were dominant, and they accounted for 75.75%, 78.85%, 85.61%, 78.89%, and 74.66% of the tags at the phylum level in the microbial communities found in the guts of IGC, PGC, ICC, HFCC, and XYCC, respectively (Figure 2b). However, the predominant phylum of Firmicutes was found among the core microbiota of fish from Dongting lake area in our study, which are consistent with Li's results (Li, Liu, et al., 2018). Interestingly, Proteobacteria was the most abundant phylum in many freshwater fish elsewhere (Wu et al., 2012; Yan et al., 2016). This finding might be due to different feeding habits and conditions. The genus *Aeromonas*, *Pseudomonas*, and *Bacteroides* were found to be dominant in majority freshwater fish intestine, followed by *Enterobacteriaceae*, *Micrococcus*, *Acinetobacter*, *Clostridium*, and others (Nayak, 2010). The rationality of gut microbiota was aided to enhancement of host's growth (Gong et al., 2019; Xie et al., 2019). It has been suggested that Proteobacteria, Firmicutes, and Bacteroidetes can play important roles in the growth of fishes. To be specific, Proteobacteria was shown to participate in the metabolism and cycling of carbon, nitrogen, and sulfur in fish (Fjellheim, Klinkenberg, Skjermo, Aasen, & Vadstein, 2010; Han et al., 2010); Bacteroidetes was found to be involved in the fermentative process and degradation of oligosaccharides (Li et al., 2015); and Firmicutes was shown to contribute to carbon metabolism (Corrigan, Leeuw, Penaud‐Frezet, Dimova, & Murphy, 2015). It was reported that the higher proportion of Firmicutes over Bacteroidetes could be associated with enhanced growth rate of fish (Li et al., 2013). Relative abundance ratios of Firmicutes over Bacteroidetes suggested that new lineage grass carp (ratio of 47.11) might have a growth advantage over Indigenous grass carp (ratio of 7.81) and that the Hefang crucian carp (ratio of 18.79) might grow faster compared with Indigenous crucian carp (ratio of 15.50) (Table S4 at https://doi.org/10.6084/m9.figshare.11389638.v2). However, the proportion in Xiangyun crucian carp (2.13) was lower than that in Indigenous crucian carp, in which the incongruence for the above conclusion might be due to the differences in fish species and the fish living conditions.

At the genus level, reads belonging to norank_f_Erysipelotrichaceae were the most abundant in all fish groups, followed by *Cetobacterium*, *Aeromonas*, etc. Several studies have identified that norank_f_Erysipelotrichaceae was responsible for digestion of a high‐fat and polysaccharides diet (Conterno, Fava, Viola, & Tuohy, 2011; Harris et al., 2014). Fish gut microbiota could promote the development of the immune system or directly activate the innate immune pathway to improve their anti‐disease ability. Meanwhile, it is important to note the norank_f_Erysipelotrichaceae, a Gram‐positive organism, may contribute to mechanisms like TLR4 pathways or enhance intestinal absorption of microbial products from other lineages, which stimulated TLR4 through expected interactions (El Kasmi et al., 2013). TLR4 was involved in innate immune defense in teleost fish, in which it could enhance resistance to diseases (Qi, Xia, Chao, Zhao, & Wu, 2017). The abundance of norank_f_Erysipelotrichaceae in PGC (36.38%, new lineage grass carp) was markedly higher than that in IGC (16.82%, Indigenous grass carp), revealing the reason why the Pure‐line grass carp has potential to hold additional disease resistance. *Cetobacterium* as a potential probiotic remained to be further studied on the intestine of fish. Among the *Cetobacterium* species, the *C. somerae* could promote the synthesis of vitamin B12 (Zhai et al., 2017). In addition, *Aeromonas*, even though sometimes pathogenic, have been detected as a necessary community member in the normal intestinal mucosa of several fishes, which could produce hydrolytic enzymes. Thus, they could act as symbionts assisting in the breakdown of dietary components (Wu et al., 2012).

As is well known, the habitat is a key factor for the survival of fish, so fish habitat differences are presumably caused by the differences in the type of food sources, including ingested bacteria, intestinal morphology and digestion, or life habits (Ni et al., 2012; Ye et al., 2014). The dietary habits considerably affect fish intestinal bacterial structure. Remarkable differences between herbivorous fish and omnivorous fish were revealed when the groups were considered separately in pairwise comparisons. Although the strains of Firmicutes dominated the gastrointestinal microbial communities in marine and freshwater fish (Jiang et al., 2019; Li, Liu, et al., 2018; Riiser et al., 2019; Wu et al., 2012), their abundance exhibited differences between herbivores and omnivore. Among marine fish, strains of Firmicutes dominated the gastrointestinal microbial communities of herbivorous fish (Egerton et al., 2018). By contrast, these bacteria were more abundant in omnivorous freshwater fish through our study. Additionally, the intestinal microbiota of grass carp harbored more cellulose‐decomposing bacteria, including *Leuconostoc*, *Clostridium*, *Ruminococcus*, and *Actinomyces* (Table S2 at https://doi.org/10.6084/m9.figshare.11389638.v2), in accordance with the cellulose‐rich plant‐based diet of herbivorous fish.

We analyzed the interaction between bacteria by construction of network graphs and highlighted the significantly abundant and relevant genera in the intestinal microbiota. The network of indigenous fish (IGC and ICC) showed lower overall connectivity, whereas the bacteria of new lineage fish groups (PGC, HFCC, and XYCC) had hubs with more node degree (Figure 4). Previous studies have shown that the microbiota structure stability declines with an increase in microbial diversity and proportion of cooperative interactions (Coyte, Schluter, & Foster, 2015). We speculated that the intestinal bacterial community was more likely to change in new lineage fish and might be regulated by changing the feeding conditions, thereby promoting the growth of new lineage fish. Further, the metabolic functional features were highlighted in the gut samples from 5 fish groups, including amino acid transport and metabolism, energy production and conversion, carbohydrate transport and metabolism, nucleotide transport and metabolism, coenzyme transport and metabolism, lipid transport and metabolism, and signal transduction mechanisms. Zhang et al. (2016) investigated a generally positive correlation between metabolic functional features and core microbiota during analysis of the Yucha samples microbial diversity by metagenomic approach. As an inherent lack of power of 16S rRNA amplicon sequencing method to resolve fine‐scaled biological complexity, we did not obtain the correlation between the predominant microbes and metabolic functional features. Hence, it would be further determined by metagenomic analysis.

Our results are the first to reveal the gut microbial composition of new lineages—grass carp and crucian carp from Dongting lake area. The observed differences of gut microbiota between new lineages and indigenous may be an important influencing factor for different growth and disease resistance.

## CONFLICT OF INTEREST

None declared.

## AUTHOR CONTRIBUTION

Sheng Zou; Conceptualization‐Supporting, Resources‐Lead, Writing‐original draft‐Equal, Writing‐review & editing‐Equal. Liang Gong; Conceptualization‐Lead, Formal analysis‐Supporting, Writing‐original draft‐Supporting, Writing‐review & editing‐Equal. Thir Ali Khan; Resources‐Equal, Writing‐review & editing‐Supporting. Lifei Pan; Resources‐Lead. Liang Yan; Resources‐Equal. Dongjie Li; Resources‐Equal. Lina Cao; Formal analysis‐Equal. Yanping Li; Formal analysis‐Equal. Xuezhi Ding; Formal analysis‐Equal. Ganfeng Yi; Writing‐review & editing‐Lead. Yunjun Sun; Formal analysis‐Equal. Shengbiao Hu; Writing‐original draft‐Lead, Writing‐review & editing‐Supporting. Liqiu Xia; Funding acquisition‐Supporting.

## ETHICS STATEMENT

This study was carried out in accordance with the recommendations of animal experimentation of the National Research Institute of Fisheries Science, Fisheries Research Agency. The protocol was approved by the Animal Care Committee of Hunan Normal University and Administration of Affairs Concerning Animal Experimentation of China.

## Data Availability

Sequence data were deposited in the NCBI Sequence Read Archive (SRA): https://www.ncbi.nlm.nih.gov/bioproject/PRJNA552740 (accession numbers: SRR9645031, SRR9645032, SRR9645033, SRR9645034, SRR9645035). Supplementary tables are available in the figshare repository at https://doi.org/10.6084/m9.figshare.11389638.v2 (Table S1: Statistics for each sample sequencing; Table S2: Taxon statistics by OTUs analysis; Table S3: COG profile of gut microbiota of each sample; Table S4: Proportion relative abundance of Firmicutes over Bacteroidetes).

## References

[mbo3996-bib-0001] Bokulich N. A. , Subramanian S. , Faith J. J. , Gevers D. , Gordon J. I. , Knight R. , … Caporaso J. G. (2013). Quality‐filtering vastly improves diversity estimates from Illumina amplicon sequencing. Nature Methods, 10, 57–59. 10.1038/nmeth.2276 23202435 PMC3531572

[mbo3996-bib-0002] Caporaso J. G. , Lauber C. L. , Walters W. A. , Berg‐Lyons D. , Huntley J. , Fierer N. , … Knight R. (2012). Ultra‐high‐throughput microbial community analysis on the Illumina HiSeq and MiSeq platforms. ISME Journal, 6, 1621–1624. 10.1038/ismej.2012.8 22402401 PMC3400413

[mbo3996-bib-0003] Caporaso J. G. , Lauber C. L. , Walters W. A. , Berg‐Lyons D. , Lozupone C. A. , Turnbaugh P. J. , … Knight R. (2011). Global patterns of 16S rRNA diversity at a depth of millions of sequences per sample. Proceedings of the National Academy of Sciences of the United States of America, 108, 4516–4522. 10.1073/pnas.1000080107 20534432 PMC3063599

[mbo3996-bib-0004] Chang, X. , Li, H. , Feng, J. , Chen, Y. , Nie, G. , & Zhang, J. (2019). Effects of cadmium exposure on the composition and diversity of the intestinal microbial community of common carp (*Cyprinus carpio* L.). Ecotoxicology and Environmental Safety, 171, 92–98. 10.1016/j.ecoenv.2018.12.066 30597321

[mbo3996-bib-0005] Chen L. , Hu C. , Lok‐Shun Lai N. , Zhang W. , Hua J. , Lam P. KS. , … Zhou B. (2018). Acute exposure to PBDEs at an environmentally realistic concentration causes abrupt changes in the gut microbiota and host health of zebrafish. Environmental Pollution, 240, 17–26. 10.1016/j.envpol.2018.04.062 29729565

[mbo3996-bib-0006] Chen S. , Wang J. , Liu S. , Qin Q. , Xiao J. , Duan W. , … Liu Y. (2009). Biological characteristics of an improved triploid crucian carp. Science China‐Life Sciences, 52, 733–738. 10.1007/s11427-009-0079-3 19727591

[mbo3996-bib-0007] Chi C. , Xue Y. , Lv N. A. , Hao Y. , Liu R. , Wang Y. , … Zhu B. (2019). Longitudinal gut bacterial colonization and its influencing factors of low birth weight infants during the first 3 months of life. Frontiers in Microbiology, 10, 1105 10.3389/fmicb.2019.01105 31156608 PMC6529567

[mbo3996-bib-0008] Conterno, L. , Fava, F. , Viola, R. , & Tuohy, K. M. (2011). Obesity and the gut microbiota: Does up‐regulating colonic fermentation protect against obesity and metabolic disease? Genes and Nutrition, 6, 241–260. 10.1007/s12263-011-0230-1 21559992 PMC3145060

[mbo3996-bib-0009] Cordova, M. S. , & Braun, C. B. (2007). The use of anesthesia during evoked potential audiometry in goldfish (*Carassius auratus*). Brain Research, 1153, 78–83. 10.1016/j.brainres.2007.03.055 17448451 PMC1952679

[mbo3996-bib-0010] Corrigan, A. , de Leeuw, M. , Penaud‐Frezet, S. , Dimova, D. , & Murphy, R. A. (2015). Phylogenetic and functional alterations in bacterial community compositions in broiler ceca as a result of mannan oligosaccharide supplementation. Applied and Environmental Microbiology, 81, 3460–3470. 10.1128/AEM.04194-14 25769823 PMC4407213

[mbo3996-bib-0011] Costello, E. K. , Gordon, J. I. , Secor, S. M. , & Knight, R. (2010). Postprandial remodeling of the gut microbiota in Burmese pythons. ISME Journal, 4, 1375–1385. 10.1038/ismej.2010.71 20520652 PMC3923499

[mbo3996-bib-0012] Coyte, K. Z. , Schluter, J. , & Foster, K. R. (2015). The ecology of the microbiome: Networks, competition, and stability. Science, 350, 663–666. 10.1126/science.aad2602 26542567

[mbo3996-bib-0013] Douterelo, I. , Sharpe, R. L. , & Boxall, J. B. (2013). Influence of hydraulic regimes on bacterial community structure and composition in an experimental drinking water distribution system. Water Research, 47, 503–516. 10.1016/j.watres.2012.09.053 23182667

[mbo3996-bib-0014] Dueñas M. , Cueva C. , Muñoz‐González I. , Jiménez‐Girón A. , Sánchez‐Patán F. , Santos‐Buelga C. , … Bartolomé B. (2015). Studies on modulation of gut microbiota by wine polyphenols: From isolated cultures to omic approaches. Antioxidants, 4, 1–21. 10.3390/antiox4010001 26785335 PMC4665564

[mbo3996-bib-0015] Edgar, R. C. (2010). Search and clustering orders of magnitude faster than BLAST. Bioinformatics, 26, 2460–2461. 10.1093/bioinformatics/btq461 20709691

[mbo3996-bib-0016] Edgar, R. C. , Haas, B. J. , Clemente, J. C. , Quince, C. , & Knight, R. (2011). UCHIME improves sensitivity and speed of chimera detection. Bioinformatics, 27, 2194–2200. 10.1093/bioinformatics/btr381 21700674 PMC3150044

[mbo3996-bib-0017] Egerton, S. , Culloty, S. , Whooley, J. , Stanton, C. , & Ross, R. P. (2018). The gut microbiota of marine fish. Frontiers in Microbiology, 9, 873 10.3389/fmicb.2018.00873 29780377 PMC5946678

[mbo3996-bib-0018] El Kasmi K. C. , Anderson A. L. , Devereaux M. W. , Vue P. M. , Zhang W. , Setchell K. D. R. , … Sokol R. J. (2013). Phytosterols promote liver injury and Kupffer cell activation in parenteral nutrition‐associated liver disease. Science Translational Medicine, 5, 206ra137 10.1126/scitranslmed.3006898 PMC4070735 24107776

[mbo3996-bib-0019] Etyemez, M. , & Balcázar, J. L. (2015). Bacterial community structure in the intestinal ecosystem of rainbow trout (*Oncorhynchus mykiss*) as revealed by pyrosequencing‐based analysis of 16S rRNA genes. Research in Veterinary Science, 100, 8–11. 10.1016/j.rvsc.2015.03.026 25843896

[mbo3996-bib-0020] Fjellheim, A. J. , Klinkenberg, G. , Skjermo, J. , Aasen, I. M. , & Vadstein, O. (2010). Selection of candidate probionts by two different screening strategies from Atlantic cod (*Gadus morhua* L.) larvae. Veterinary Microbiology, 144, 153–159. 10.1016/j.vetmic.2009.12.032 20097491

[mbo3996-bib-0021] Food and Agriculture Organisation of the United Nations [FAO] (2016). Rome, Italy: FAO; p. 200.

[mbo3996-bib-0022] Gao C.‐H. , Mortimer M. , Zhang M. , Holden P. A. , Cai P. , Wu S. , … Huang Q. (2019). Impact of metal oxide nanoparticles on in vitro DNA amplification. PeerJ, 7, e7228 10.7717/peerj.7228 31293839 PMC6599668

[mbo3996-bib-0023] Gong L. , He H. , Li D. , Cao L. , Khan T. A. , Li Y. , … Xia L. (2019). A new isolate of *Pediococcus pentosaceus* (SL001) with antibacterial activity against fish pathogens and potency in facilitating the immunity and growth performance of grass carps. Frontiers in Microbiology, 10, 1384 10.3389/fmicb.2019.01384 31316478 PMC6610308

[mbo3996-bib-0024] Grond, K. , Lanctot, R. B. , Jumpponen, A. , & Sandercock, B. K. (2017). Recruitment and establishment of the gut microbiome in Arctic shorebirds. FEMS Microbiology Ecology, 93, fix142. 10.1093/femsec/fix142 29069418

[mbo3996-bib-0025] Han S. , Liu Y. , Zhou Z. , He S. , Cao Y. , Shi P. , … Ringø E. (2010). Analysis of bacterial diversity in the intestine of grass carp (*Ctenopharyngodon idellus*) based on 16S rDNA gene sequences. Aquaculture Research, 42, 47–56. 10.1111/j.1365-2109.2010.02543.x

[mbo3996-bib-0026] Harris J. K. , El Kasmi K. C. , Anderson A. L. , Devereaux M. W. , Fillon S. A. , Robertson C. E. , … Sokol R. J. (2014). Specific microbiome changes in a mouse model of parenteral nutrition associated liver injury and intestinal inflammation. PLoS ONE, 9, e110396 10.1371/journal.pone.0110396 25329595 PMC4203793

[mbo3996-bib-0027] Jiang, Y. , Wang, Y. , Zhang, Z. , Liao, M. , Li, B. , Rong, X. , & Chen, G. (2019). Responses of microbial community structure in turbot (*Scophthalmus maximus*) larval intestine to the regulation of probiotic introduced through live feed. PLoS ONE, 14, e0216590 10.1371/journal.pone.0216590 31067264 PMC6505946

[mbo3996-bib-0028] Klemetsen, T. , Willassen, N. P. , & Karlsen, C. R. (2019). Full‐length 16S rRNA gene classification of Atlantic salmon bacteria and effects of using different 16S variable regions on community structure analysis. Microbiologyopen, 8, e898 10.1002/mbo3.898 31271529 PMC6813439

[mbo3996-bib-0029] Li, H. , Zhong, Q. P. , Wirth, S. , Wang, W. W. , Hao, Y. T. , Wu, S. G. , … Wang, G. (2015). Diversity of autochthonous bacterial communities in the intestinal mucosa of grass carp (*Ctenopharyngodon idellus*) (Valenciennes) determined by culture‐dependent and culture‐independent techniques. Aquaculture Research, 46, 2344–2359.

[mbo3996-bib-0030] Li W. , Liu J. , Tan H. , Yang C. , Ren L. I. , Liu Q. , … Liu S. (2018). Genetic effects on the gut microbiota assemblages of hybrid fish from parents with different feeding habits. Frontiers in Microbiology, 9, 2972 10.3389/fmicb.2018.02972 30564218 PMC6288232

[mbo3996-bib-0031] Li, X. , Yan, Q. , Xie, S. , Hu, W. , Yu, Y. , & Hu, Z. (2013). Gut microbiota contributes to the growth of fast‐growing transgenic common carp (*Cyprinus carpio* L.). PLoS ONE, 8, e64577 10.1371/journal.pone.0064577 23741344 PMC3669304

[mbo3996-bib-0032] Li, X. , Zhu, Y. , Ringo, E. , Wang, X. , Gong, J. , & Yang, D. (2018). Intestinal microbiome and its potential functions in bighead carp (*Aristichthys nobilis*) under different feeding strategies. PeerJ, 6, e6000. 30533302 10.7717/peerj.6000 PMC6283038

[mbo3996-bib-0033] Lin, W. , Li, L. , Chen, J. , Li, D. P. , Hou, J. , Guo, H. H. , & Shen, J. Z. (2018). Long‐term crowding stress causes compromised nonspecific immunity and increases apoptosis of spleen in grass carp (*Ctenopharyngodon idella*). Fish & Shellfish Immunology, 80, 540–545. 10.1016/j.fsi.2018.06.050 29964198

[mbo3996-bib-0034] Liu Q. , Qi Y. , Liang Q. , Song J. , Liu J. , Li W. , … Liu S. (2019). Targeted disruption of tyrosinase causes melanin reduction in *Carassius auratus cuvieri* and its hybrid progeny. Science China‐Life Sciences, 62, 1194–1202. 10.1007/s11427-018-9404-7 30593611

[mbo3996-bib-0035] Liu S. , Liu Y. , Zhou G. , Zhang X. , Luo C. , Feng H. , … Yang H. (2001). The formation of tetraploid stocks of red crucian carp×common carp hybrids as an effect of interspecific hybridization. Aquaculture, 192, 172–186. 10.1016/S0044-8486(00)00451-8

[mbo3996-bib-0036] Liu, X. , Sha, Z. , Wang, C. , Li, D. , & Bureau, D. P. (2018). A web‐based combined nutritional model to precisely predict growth, feed requirement and waste output of gibel carp (*Carassius auratus gibelio*) in aquaculture operations. Aquaculture, 492, 335–348. 10.1016/j.aquaculture.2018.04.019

[mbo3996-bib-0037] Liu, Y. , Yao, Y. , Li, H. , Qiao, F. , Wu, J. , Du, Z. Y. , & Zhang, M. (2016). Influence of endogenous and exogenous estrogenic endocrine on intestinal microbiota in zebrafish. PLoS ONE, 11, e0163895 10.1371/journal.pone.0163895 27701432 PMC5049800

[mbo3996-bib-0038] Magoc, T. , & Salzberg, S. L. (2011). FLASH: Fast length adjustment of short reads to improve genome assemblies. Bioinformatics, 27, 2957–2963. 10.1093/bioinformatics/btr507 21903629 PMC3198573

[mbo3996-bib-0039] Mao, Z. W. , Fu, Y. Q. , Wang, Y. D. , Wang, S. , Zhang, M. H. , Gao, X. , & Liu S. (2019). Evidence for paternal DNA transmission to gynogenetic grass carp. BMC Genetics, 20, 3. 30616510 10.1186/s12863-018-0712-x PMC6323743

[mbo3996-bib-0040] Mardis, E. , & McCombie, W. R. (2017). Library quantification using PicoGreen fluorometry. Cold Spring Harbor Protocols, 2017(5), pdb.prot094722. 10.1101/pdb.prot094722 27803277

[mbo3996-bib-0041] Nayak, S. K. (2010). Role of gastrointestinal microbiota in fish. Aquaculture Research, 41, 1553–1573. 10.1111/j.1365-2109.2010.02546.x

[mbo3996-bib-0042] Ni, J. J. , Yu, Y. H. , Zhang, T. L. , & Gao, L. (2012). Comparison of intestinal bacterial communities in grass carp, *Ctenopharyngodon idellus*, from two different habitats. Chinese Journal of Oceanology and Limnology, 30, 757–765. 10.1007/s00343-012-1287-4

[mbo3996-bib-0043] Qi, D. , Xia, M. , Chao, Y. , Zhao, Y. , & Wu, R. (2017). Identification, molecular evolution of toll‐like receptors in a Tibetan schizothoracine fish (*Gymnocypris eckloni*) and their expression profiles in response to acute hypoxia. Fish & Shellfish Immunology, 68, 102–113. 10.1016/j.fsi.2017.07.014 28698123

[mbo3996-bib-0044] Qin, Y. , Hou, J. , Deng, M. , Liu, Q. , Wu, C. , Ji, Y. , & He, X. (2016). Bacterial abundance and diversity in pond water supplied with different feeds. Scientific Reports, 6, 35232 10.1038/srep35232 27759010 PMC5069485

[mbo3996-bib-0045] Quast, C. , Pruesse, E. , Yilmaz, P. , Gerken, J. , Schweer, T. , Yarza, P. , … Glöckner F. O. (2013). The SILVA ribosomal RNA gene database project: Improved data processing and web‐based tools. Nucleic Acids Research, 41, D590–D596. 23193283 10.1093/nar/gks1219 PMC3531112

[mbo3996-bib-0046] Ray, A. K. , Ghosh, K. , & Ringo, E. (2012). Enzyme‐producing bacteria isolated from fish gut: A review. Aquaculture Nutrition, 18, 465–492. 10.1111/j.1365-2095.2012.00943.x

[mbo3996-bib-0047] Riiser, E. S. , Haverkamp, T. H. A. , Varadharajan, S. , Borgan, O. , Jakobsen, K. S. , Jentoft, S. , & Star, B. (2019). Switching on the light: Using metagenomic shotgun sequencing to characterize the intestinal microbiome of Atlantic cod. Environmental Microbiology, 21, 2576–2594. 10.1111/1462-2920.14652 31091345

[mbo3996-bib-0048] Siriyappagouder, P. , Galindo‐Villegas, J. , Lokesh, J. , Mulero, V. , Fernandes, J. M. O. , & Kiron, V. (2018). Exposure to yeast shapes the intestinal bacterial community assembly in zebrafish Larvae. Frontiers in Microbiology, 9, 1868 10.3389/fmicb.2018.01868 30154775 PMC6103253

[mbo3996-bib-0049] Song W. , Li L. , Huang H. , Jiang K. , Zhang F. , Chen X. , … Ma L. (2016). The gut microbial community of antarctic fish detected by 16S rRNA gene sequence analysis. BioMed Research International, 2016, 3241529 10.1155/2016/3241529 27957494 PMC5124462

[mbo3996-bib-0050] Walter, J. M. , Bagi, A. , & Pampanin, D. M. (2019). Insights into the potential of the Atlantic cod gut microbiome as biomarker of oil contamination in the marine environment. Microorganisms, 7, E209 10.3390/microorganisms7070209 31336609 PMC6680985

[mbo3996-bib-0051] Wang, Q. , Garrity, G. M. , Tiedje, J. M. , & Cole, J. R. (2007). Naive Bayesian classifier for rapid assignment of rRNA sequences into the new bacterial taxonomy. Applied and Environmental Microbiology, 73, 5261–5267. 10.1128/AEM.00062-07 17586664 PMC1950982

[mbo3996-bib-0052] Wang S. , Tang C. , Tao M. , Qin Q. , Zhang C. , Luo K. , … Liu S. (2019). Establishment and application of distant hybridization technology in fish. Science China‐Life Sciences, 62, 22–45. 10.1007/s11427-018-9408-x 30554295

[mbo3996-bib-0053] Wong, K. , Shaw, T. I. , Oladeinde, A. , Glenn, T. C. , Oakley, B. , & Molina, M. (2016). Rapid microbiome changes in freshly deposited cow feces under field conditions. Frontiers in Microbiology, 7, 500 10.3389/fmicb.2016.00500 27148189 PMC4830129

[mbo3996-bib-0054] Wu, P. , Mo, W. , Wang, Y. , Wu, Y. , Zhang, Y. , Chen, Z. , & Li, N. (2019). Effluent containing *Rubrivivax gelatinosus* promoting the yield, digestion system, disease resistance, mTOR and NF‐kB signaling pathway, intestinal microbiota and aquaculture water quality of crucian carp. Fish & Shellfish Immunology, 94, 166–174. 10.1016/j.fsi.2019.08.015 31446081

[mbo3996-bib-0055] Wu, S. , Wang, G. , Angert, E. R. , Wang, W. , Li, W. , & Zou, H. (2012). Composition, diversity, and origin of the bacterial community in grass carp intestine. PLoS ONE, 7, e30440 10.1371/journal.pone.0030440 22363439 PMC3282688

[mbo3996-bib-0056] Xiao, J. , Fu, Y. , Wu, H. , Chen, X. , Liu, S. , & Feng, H. (2019). MAVS of triploid hybrid of red crucian carp and allotetraploid possesses the improved antiviral activity compared with the counterparts of its parents. Fish & Shellfish Immunology, 89, 18–26. 10.1016/j.fsi.2019.03.044 30905838

[mbo3996-bib-0057] Xie J.‐J. , Liu Q.‐Q. , Liao S. , Fang H.‐H. , Yin P. , Xie S.‐W. , … Niu J. (2019). Effects of dietary mixed probiotics on growth, non‐specific immunity, intestinal morphology and microbiota of juvenile pacific white shrimp, *Litopenaeus vannamei* . Fish & Shellfish Immunology, 90, 456–465. 10.1016/j.fsi.2019.04.301 31075403

[mbo3996-bib-0058] Yan Q. , Li J. , Yu Y. , Wang J. , He Z. , Van Nostrand J. D. , … Zhou J. (2016). Environmental filtering decreases with fish development for the assembly of gut microbiota. Environmental Microbiology, 18, 4739–4754. 10.1111/1462-2920.13365 27130138

[mbo3996-bib-0059] Yang S. , Duan Y. , Zhang J. , Zhou J. , Liu Y. A. , Du J. , … Han S. (2017). Observational comparisons of intestinal microbiota characterizations, immune enzyme activities, and muscle amino acid compositions of loach in paddy fields and ponds in Sichuan Province. Applied Microbiology and Biotechnology, 101, 4775–4789. 10.1007/s00253-017-8167-y 28210795

[mbo3996-bib-0060] Ye, L. , Amberg, J. , Chapman, D. , Gaikowski, M. , & Liu, W. T. (2014). Fish gut microbiota analysis differentiates physiology and behavior of invasive Asian carp and indigenous American fish. ISME Journal, 8, 541–551. 10.1038/ismej.2013.181 24132079 PMC3930320

[mbo3996-bib-0061] Zhai Q. , Yu L. , Li T. , Zhu J. , Zhang C. , Zhao J. , … Chen W. (2017). Effect of dietary probiotic supplementation on intestinal microbiota and physiological conditions of Nile tilapia (*Oreochromis niloticus*) under waterborne cadmium exposure. Antonie Van Leeuwenhoek, 110, 501–513. 10.1007/s10482-016-0819-x 28028640

[mbo3996-bib-0062] Zhang H. , Liu S. , Zhang C. , Tao M. , Peng L. , You C. , … Liu Y. (2011). Induced gynogenesis in grass carp (*Ctenopharyngodon idellus*) using irradiated sperm of allotetraploid hybrids. Marine Biotechnology, 13, 1017–1026. 10.1007/s10126-011-9365-8 21279407

[mbo3996-bib-0063] Zhang J. , Wang X. , Huo D. , Li W. U. , Hu Q. , Xu C. , … Li C. (2016). Metagenomic approach reveals microbial diversity and predictive microbial metabolic pathways in Yucha, a traditional Li fermented food. Scientific Reports, 6, 32524 10.1038/srep32524 27578483 PMC5006176

